# Biatrial versus Isolated Left Atrial Ablation in Atrial Fibrillation: A Systematic Review and Meta-Analysis

**DOI:** 10.1155/2018/3651212

**Published:** 2018-04-29

**Authors:** Hongmu Li, Xifeng Lin, Xun Ma, Jun Tao, Rongjun Zou, Songran Yang, Haibo Liu, Ping Hua

**Affiliations:** ^1^Department of Cardiovascular Surgery, Quanzhou First Hospital of Fujian Medical University, Quanzhou 362000, China; ^2^Department of Cardiovascular Surgery, Sun Yat-sen Memorial Hospital, Sun Yat-sen University, Guangzhou 510120, China; ^3^Guangzhou Women and Children's Medical Center, Guangzhou 510623, China; ^4^The Biobank of Sun Yat-sen Memorial Hospital, Sun Yat-sen University, Guangzhou 510120, China; ^5^Guangdong Province Key Laboratory of Brain Function and Disease, Zhongshan School of Medicine, Sun Yat-sen University, Guangzhou 510080, China

## Abstract

**Objective:**

The outcomes of biatrial ablation (BA) and isolated left atrial ablation (LA) in atrial fibrillation remain inconclusive. In this meta-analysis, we assess the currently available evidence to compare outcomes between BA and LA.

**Methods:**

Electronic searches were performed from database inception to December 2016, and relevant studies were accessed. Odds ratios and weight mean differences with 95% confidence intervals are reported. Twenty-one studies comprising 3609 patients were included in the present meta-analysis.

**Results:**

The prevalence of sinus rhythm in the BA cohort was similar to that in the LA cohort at discharge, at 12 months, and after more than 1 year of follow-up. However, at 6 months, the prevalence of sinus rhythm was higher in the BA cohort than in the LA cohort. The rate of permanent pacemaker implantation was higher in the BA cohort than in the LA cohort. However, 30-day and late mortality and neurological events were similar between the BA and LA groups.

**Conclusion:**

There was no significant difference in the rate of restored sinus rhythm, the risk of death, and cerebrovascular events between BA and LA, but BA had a higher rate of permanent pacemaker implantation.

## 1. Introduction 

Atrial fibrillation (AF) is a major healthcare problem worldwide which has enormous economic and public health implications. AF is associated with an increased risk of stroke, heart failure, and all-cause mortality [1–3].

Surgical ablation was introduced as a treatment option by Cox et al. [4] in 1991, and it is currently an effective curative strategy for AF. Haïssaguerre et al. [5] suggested that ectopic beats from pulmonary veins may cause AF, and the field of pulmonary vein isolation (PVI) was consequently established and is now performed via catheter or surgical ablation [6]. Left ablation (LA) has historically been the main method used to treat AF, and it has a fairly good clinical effect [7, 8]. However, some studies have suggested that LA is less efficacious than biatrial ablation (BA), especially when a right-side AF trigger is present [9]. Hence, because outcomes have been inconclusive, Phan et al. [10] and Zheng et al. [11] reported relevant meta-analyses in 2014, but they arrived at a different conclusion. The authors showed that BA was more effective than LA and that the rate of permanent pacemaker implantation was also higher in the BA cohort than in the LA cohort. However, Zheng et al. [11] suggested that the effects of BA and LA are the same.

In the past two years, several other studies [8, 12–15] have compared BA and LA in AF, with controversial outcomes. Hence, in this meta-analysis, we sought to assess the current evidence available on this issue.

## 2. Methods

### 2.1. Literature Search Strategy

**Figure 1:**
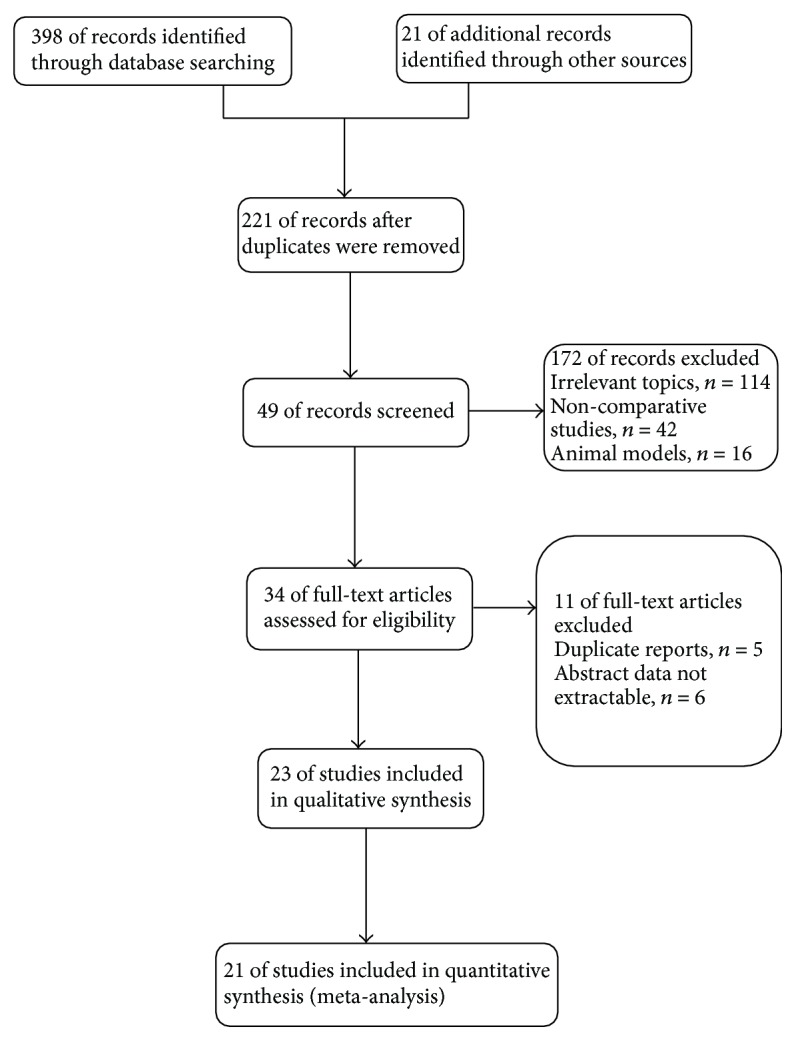
Selection of studies for the meta-analysis.

Electronic searches were performed in August 2016 without search restrictions. The primary sources were the electronic Medline, PubMed, Cochrane Library, and EMBASE databases, which were searched from their date of inception to August 2016. The following search terms were used: “maze,” “biatrial,” “bi-atrial,” “uniatrial,” “left atrial,” and “ablation.” When duplicate published trials with accumulating numbers of patients or increased lengths of follow-up were encountered, the most recent or most complete report was considered. All titles and abstracts identified in the electronic search were uploaded into an EndNote (version X7; Thomson Corporation, Stanford, USA) database (Figure 1).

### 2.2. Inclusion and Exclusion Criteria

All available randomized, controlled trials (RCTs) and retrospective comparative studies that compared BA with LA in all age groups were included. Abstracts, case reports, conference presentations, editorials, reviews, and expert opinions were excluded. When institutions published duplicate studies with accumulating numbers of patients or increased lengths of follow-up, only the most complete report was included. Reference lists were also manually searched for further relevant studies.

### 2.3. Data Extraction and Critical Appraisal

Two reviewers (Hongmu Li and Xifeng Lin) conducted data extraction independent of the included studies. Data on authorship, year of publication, study design, study population, baseline characteristics, characteristics related to outcomes, and duration of follow-up were extracted from each study. Reported percentages were approximated to numbers. The risk of bias was assessed using the Downs and Black checklist [16] for randomized and observational studies. Discrepancies between the reviewers were resolved by discussion until consensus was reached. The final results were reviewed by the senior investigator (Ping Hua).

### 2.4. Quality Assessment and Statistical Analysis

The included studies were rated to determine the level of quality of the provided evidence according to the criteria of the Centre for Evidence-Based Medicine in Oxford, UK [17]. The methodological quality of the RCTs was assessed with the Cochrane risk of bias tool [18]. The methodological quality of retrospective studies was assessed with the modified Newcastle-Ottawa scale [19, 20].

This meta-analysis was performed using Review Manager Version 5.3 (Cochrane Collaboration, Oxford, UK). Dichotomous variables from individual studies were analyzed using odds ratios (ORs) with 95% confidence intervals (CIs). *Q*-statistics (*P* < 0.10) or* I*^2^ statistics were performed to test for heterogeneity between included studies, and values of 50% or higher were considered to be indicative of substantial heterogeneity. If there was substantial heterogeneity, the possible clinical and methodological reasons for this were explored qualitatively. Publication bias was examined through a visual inspection of funnel plots and assessed by applying Egger's weighted regression statistic and considering a *P* value less than 0.05 as indicating significant publication bias. A *P* value of less than 0.05 was considered statistically significant for all analyses.

### 2.5. Synthesis of Evidence

Our electronic literature search resulted in the retrieval of 398 citations. Of these, 372 were excluded after duplicate and irrelevant references were excluded, and 49 potentially relevant articles were retrieved. Finally, following a manual search of reference lists and a critical appraisal, 21 studies comprising 3609 patients were included in this meta-analysis. Two articles had redundant publications but covered different characteristics [21, 22].

### 2.6. Quality Assessment and Baseline Characteristics of Eligible Studies

**Figure 2:**
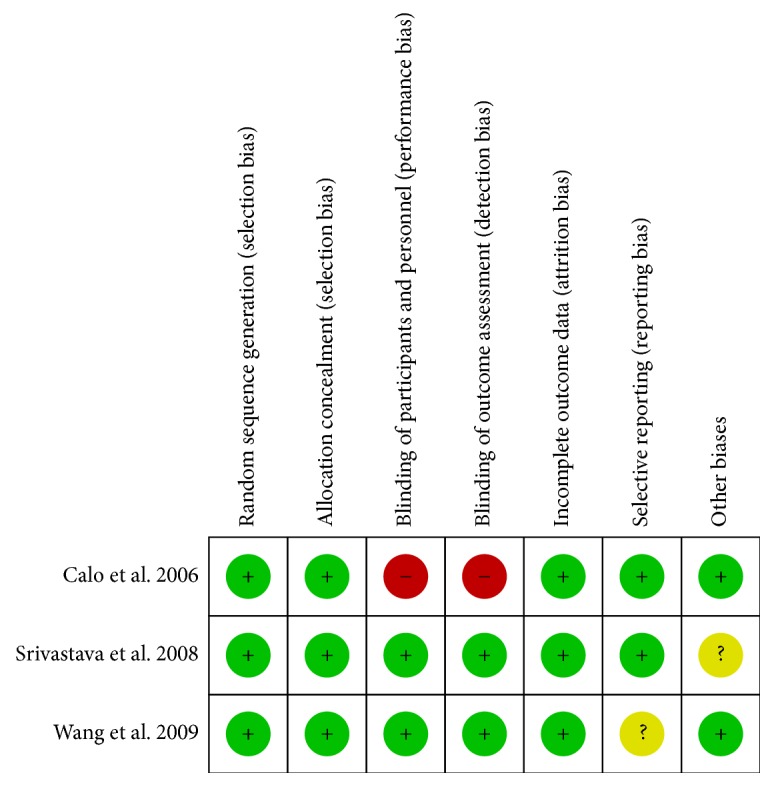
Risk of bias in RCT studies.

In all, 21 studies were included in this meta-analysis, including three prospective randomized trials [23–25], five prospective observational studies [13, 26–29], and 13 retrospective observational studies [8, 12, 14, 15, 21, 22, 30–36]. The risk of bias in each study is shown in Figure 2 and Table 1. Among the 3609 patients, 1901 received BA, and 1708 received LA. Patients in the BA group underwent a classical or modified maze procedure, including both left-sided and right-sided maze procedures. However, patients in the LA group underwent a left-sided maze procedure that included PVI, left atrial posterior wall isolation, mitral isthmus ablation, and left atrial appendage excision.

Three studies used cryoablation energy [15, 27, 31], eleven studies used radiofrequency energy [12–14, 21, 23, 25, 26, 29, 32–34], and the remaining studies used a combination of different energy sources, including radiofrequency, cryoablation, and microwave and “cut-and-sew” [8, 22, 24, 28, 30, 35, 36]. Concomitant coronary artery bypass grafting surgery was reported in 10 studies [8, 13, 21, 22, 25, 27–31, 36], while a concomitant valvular operation was performed in other included studies. The baseline characteristics of the patients in the included studies are shown in Tables 2 and 3.

## 3. Outcomes

### 3.1. Assessment of Efficacy

**Figure 3:**
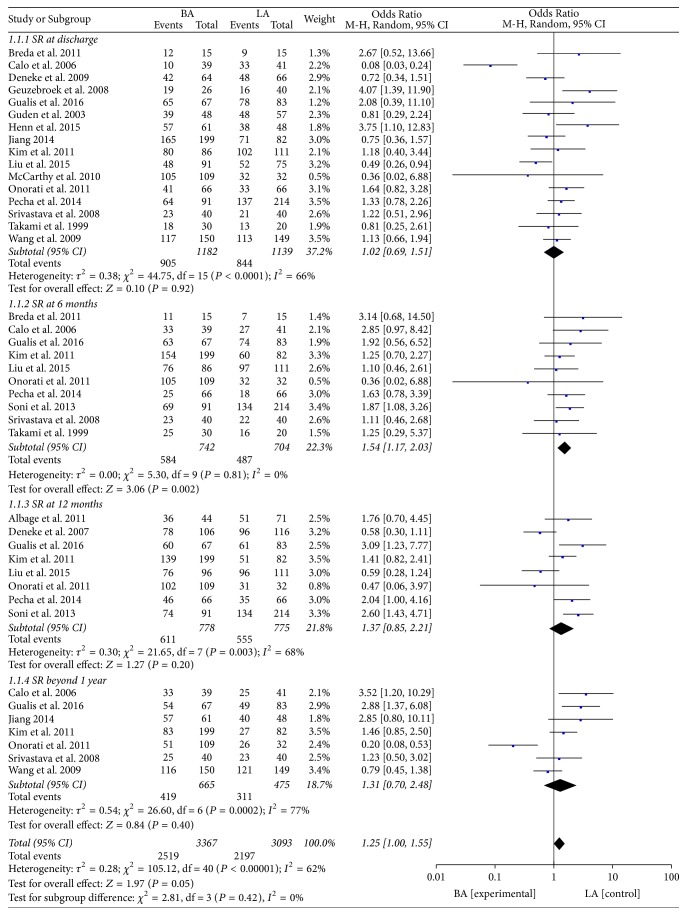
Restored SR at discharge, 6 months, and 12 months and beyond 1 year.

The data were pooled from 16 studies [12–15, 21–26, 28, 29, 31–33, 36] that assessed the efficacy of restoring sinus rhythm (SR), and the results showed that there was no significant difference between the BA and LA groups at discharge (78.3% versus 73.86%; OR: 1.02; 95% CI: 0.69–1.51; *P* = 0.92;* I*^2^ = 66%). However, the overall prevalence of SR was higher in the BA group than in the LA group at a 6-month follow-up (78.82% versus 69.67%; OR: 1.54; 95% CI: 1.17–2.03; *P* = 0.002;* I*^2^ = 0%) [14, 15, 22, 24–26, 30–32, 36]. For patients with a follow-up at 12 months [8, 14, 15, 26, 27, 30, 31, 34] and after more than 1 year [12, 15, 23–25, 31], the prevalence of SR in the BA group was similar to that in the LA group (63.01% versus 65.47%; OR: 1.31; 95% CI: 0.70–2.48; *P* = 0.40;* I*^2^ = 77%). The weighted average mean follow-up for studies reporting SR after more than 1 year was 23.3 months. These results are shown in Figure 3.

### 3.2. Mortality and Major Complications

**Figure 4:**
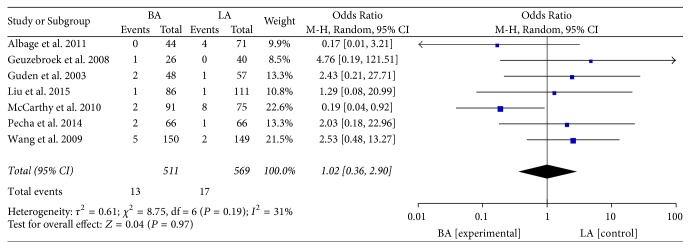
Mortality within 30 days.

**Figure 5:**
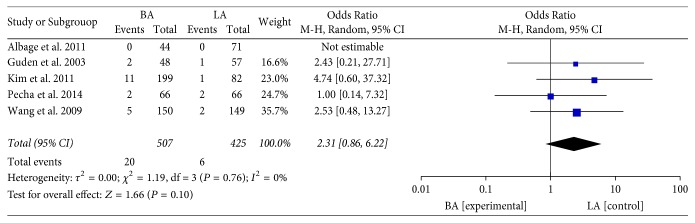
Late mortality.

Eight studies with 1185 patients investigated mortality after the BA or LA procedure. When effects were pooled, there was no significant difference in either early mortality [8, 14, 23, 27–29, 33] (<30 days, OR: 1.02; 95% CI: 0.36–2.90; *P* = 0.97;* I*^2^ = 31%, Figure 4) or late mortality [23, 27, 29, 31, 35] (OR: 2.31; 95% CI: 0.86–6.22; *P* = 0.10;* I*^2^ = 0%, Figure 5) between the BA and LA groups.

**Figure 6:**
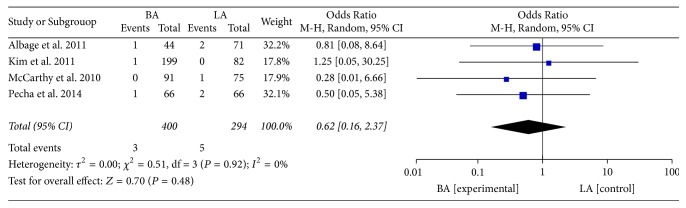
Cerebrovascular events.

**Figure 7:**
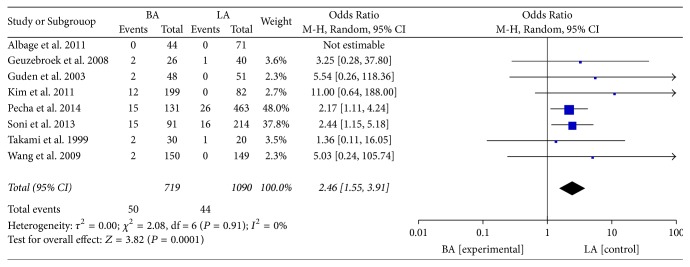
Permanent pacemaker implantation.

There was no significant increase in the risk of cerebrovascular events [8, 27, 28, 31] between the two groups (OR: 0.61; 95% CI: 0.16–2.40; *P* = 0.48;* I*^2^ = 0%, Figure 6). In eight studies that compared LA with BA, BA increased the risk of permanent pacemaker implantation [22, 23, 30, 31, 33, 36] (OR: 2.46; 95% CI: 1.55–3.91; *P* = 0.0001;* I*^2^ = 0%, Figure 7). No heterogeneity was observed.

### 3.3. Sensitivity Analysis and Publication Bias

The risk of bias was comprehensively assessed according to the guidelines of the Cochrane Collaboration, and neither visual inspection of funnel plots nor Egger's test detected significant publication bias for the major outcomes explored in this meta-analysis, including the prevalence of SR at discharge (*t* = 0.04; *P* = 0.972), SR at a 6-month follow-up (*t* = 0.27; *P* = 0.791), SR at a 12-month follow-up (*t* = 0.90; *P* = 0.401), SR after more than 1 year (*t* = 0.52; *P* = 0.626), early mortality (*t* = 1.03; *P* = 0.363), late mortality (*t* = −1.07; *P* = 0.397), neurological events (*t* = 51.13; *P* = 0.012), and permanent pacemaker implantation (*t* = 2.42; *P* = 0.060). To evaluate the effect of heterogeneity on the pooled effect, we carried out a sensitivity analysis. Sensitivity and subgroup analyses found no significant heterogeneity (Table 4).

## 4. Discussion

Two recent meta-analyses [10, 11] that compared BA, LA, and surgical ablation in AF arrived at conflicting conclusions. However, these meta-analyses excluded several studies that compared BA with LA [8, 12–15, 25]. Therefore, we performed a new meta-analysis to compare BA with LA. This meta-analysis included three RCTs and 18 retrospective studies that collectively contained 3609 patients and compared the efficacy and safety of BA and LA. There was no significant difference between BA and LA in the rate of restored SR, but BA groups had a higher probability of SR after 6 months of follow-up. We also found that while BA and LA had similar rates of death and cerebrovascular events, the BA groups had a higher rate of permanent pacemaker implantation.

A pooled analysis of restored postoperative SR showed that there was no difference between the BA and LA groups. However, several recent studies [21, 23, 36] have shown that BA is inferior to the more complete LA when used alone. Patients in the LA group had shorter aortic cross-clamping times and cardiopulmonary bypass times than were observed in the BA group. Furthermore, the techniques used in AF ablation vary widely, even within the same procedure group, and if the different lesion sets used for ablation were included, the results may have indicated that this procedure has greater efficacy.

In contrast, some studies have reported that BA is superior to LA for restoring SR [8, 12, 15, 25–28, 30–33]. This finding rests mainly on the finding that BA groups have much more tissue damage and a higher rate of cardiac conduction system injury. However, all of these studies have common limitations. First, some patients took antiarrhythmic drugs (including amiodarone) perioperatively and continued the use of these drugs until the operation, and few researchers sufficiently addressed this variable. Second, the sample size in most of the articles was small (less than 150 individuals), weakening the power of the studies. Third, long rhythm registration during follow-up was not available in all of the patients. Furthermore, only a few of the studies were RCTs. Unlike previous reviews, we included the largest studies in our meta-analysis, and our inclusion criteria did not limit our search to articles published in English. We also conducted a subgroup analysis of RCT and non-RCT studies and of small-sample and large-sample studies to assess the effect of heterogeneity on the pooled effect estimate.

The findings of the present meta-analysis confirm that BA increases the risk of permanent pacemaker implantation. This finding may be attributed to the fact that LA has shorter aortic cross-clamping and cardiopulmonary bypass times and promotes more extensive lesions. There was no significant increase in the risk of cerebrovascular events or early and late mortality between the two groups. We hypothesize that report selection resulted in fewer such events, and these results remain to be discussed.

The most important findings of our meta-analysis include the following: (1) LA and BA were equally effective in restoring SR, (2) BA resulted in higher prevalence of SR at the 6-month follow-up, and (3) unlike previous analyses, this meta-analysis included the largest studies, and its inclusion criteria did not limit the search to articles published in the English language.

The results of our study show that there was no difference in the rate of restored SR between LA and BA. While some previous studies have proposed that BA alone is inferior to a more complete LA, this significance disappeared in a multivariate analysis. The difference in these results may have been caused by differences in inclusion criteria between previous studies and our study. The other studies limited inclusion to articles reported in the English language. Additionally, the techniques used for AF ablation varied widely, including, for example, the use of different lesion sets, even within same procedure group.

One of the most important reasons that researchers have suggested for why BA is better than LA at restoring SR is that there is a significant difference in electrical activity between patients with chronic and paroxysmal AF [37, 38]. Lazar et al. [37] demonstrated that a left-to-right atrial frequency gradient exists in paroxysmal but not persistent AF. This prompted them to propose that the maintenance of persistent or chronic AF may be less dependent on the posterior left atrium. Additionally, Sanders et al. [39] proposed that, in patients with paroxysmal AF, the dominant sources of activity are often localized in the pulmonary veins. In contrast, in patients with permanent AF, the dominant sites are more often localized in the atria, including right atrial sites. Unsurprisingly, patients with persistent or long-standing persistent AF are more likely to receive BA, and this may affect clinical outcomes [25].

The present meta-analysis has the following limitations. Its main limitation is that only three small-sample RCTs were included. Inadequate random sequence generation and blinding tend to increase the risk of bias. Hence, larger RCTs are needed to determine the best treatment. Another limitation is that the original meta-analysis was based on the assumption that the surgical subgroups (BA and LA) were sufficiently similar to be assessed together, but the operation methods and ablation technologies used in these procedures are continually developing. Additionally, there was extreme heterogeneity among the studies in study design, data, and energy source, and a subgroup analysis yielded results that differed from those obtained in the original analysis. Future systematic reviews should, when sufficient literature is available, evaluate different indications separately. Finally, follow-up periods were generally short. Therefore, the long-term outcomes of BA and LA remain to be explored.

## 5. Conclusion

In this comparative meta-analysis, we show that BA is not more efficacious than LA in restoring SR. Additionally, the risks of death and cerebrovascular events are significantly different between BA and LA, but BA results in a higher rate of permanent pacemaker implantation.

## Figures and Tables

**Table 1:** Risk of bias in observational studies.

Study	Selection			Comparability		Outcome		Quality score
	Assignment for treatment	Representative treatment group	Representative reference group	Comparable for 1, 2, 3, 4	Comparable for 5, 6, 7, 8	Assessment of outcome	Adequate follow-up	
Gualis et al. 2016	No	Yes	Yes	1, 2	5, 8	Yes	No	★★★★★
Liu et al., 2015	No	Yes	Yes	1, 2	5, 8	No	Yes	★★★★★
Henn et al., 2015	No	Yes	Yes	NR	5, 6	Yes	No	★★★★
Pecha et al., 2014	No	Yes	Yes	1, 2	5, 8	Yes	NR	★★★★★
Jiang, 2014	No	Yes	Yes	1, 2	8	Yes	No	★★★★
Soni et al., 2013	No	Yes	Yes	1, 2	8	No	No	★★★
Pecha et al., 2014	No	Yes	Yes	1, 2	5, 6	Yes	Yes	★★★★★★
Onorati et al., 2011	No	Yes	Yes	1, 2	5, 6, 8	Yes	Yes	★★★★★★★
Kim et al., 2011	No	Yes	Yes	1, 2	7, 8	Yes	No	★★★★★
Breda et al., 2011	No	Yes	Yes	1, 2	6, 8	No	No	★★★★
Albage et al., 2011	No	Yes	Yes	1, 2	7	Yes	No	★★★★
McCarthy et al., 2010	No	Yes	Yes	1, 2	8	Yes	Yes	★★★★★★
Deneke et al., 2009	No	Yes	Yes	1, 4	6, 8	Yes	Yes	★★★★★★
Geuzebroek et al., 2008	No	Yes	Yes	1, 2	5	No	Yes	★★★★
Deneke et al., 2007	No	Yes	Yes	1, 4	8	Yes	Yes	★★★★★
Ryan et al., 2004	No	Yes	Yes	NR	5, 6, 7, 8	Yes	No	★★★★★
Guden et al., 2003	No	Yes	Yes	1, 2, 3	NR	Yes	Yes	★★★★★
Takami et al., 1999	No	Yes	Yes	1, 2, 3	5, 7, 8	Yes	Yes	★★★★★★★

NR: no report; comparability variables: 1 = age; 2 = gender; 3 = body mass index; 4 = type of AF; 5 = Euro score; 6 = preoperative antiarrhythmic drugs; 7 = anatomic complexity (more than one artery and/or vein); 8 = single surgeon.

**Table 2:** Characteristics of the studies that were initially included in the meta-analysis.

First author	Year	Country	Study period	Type of study	Follow-up (MO)	*n* (BA)	*n* (LA)	Type of ablation	Lesion set	Cardiac operation
Gualis	2016	Spain	2006–2011	R	36	67	83	CY	PVI, LAA, RAA, TC, CS, WG	AVR AVP MVR MVP TVP

Liu	2015	China	2012-2012	R	3–12R	86	111	RF	PVI, LAA, RAA	MVR DVR TVP

Henn	2015	USA	2002–2014	P, non-RCT	5 years	532	44	RF	PVI, LAA, RAA, TC, CS, WG	AVR CABG MVR MVP TVP

Pecha	2014	Germany	2008–2011	R	12	66	66	RF, CY	LVI, LAA, CI, RAA, TC	CABG AVR MVR TVR

Jiang	2014	China	2008–2012	R	NR	61	48	RF	PVI, MV, LAA, RAA, TC, CS, WG	MVR MVP TVP

Soni	2013	USA	2007–2011	R	12	91	214	RF, CY, MW	PVI, PW, MI, LAA MM	AVR MVR CABG MVP TVP

Pecha	2014	Germany	2003–2012	R	30 days	131	463	CY, RF	LVI, LAA, BLI, CI, RAA, TC	AVR AVP MVR MVP TVP CABG ASD VSD

Onorati	2011	Italy	2003–2008	P, non-RCT	15	109	32	RF	PVI MV LAA RAA TC CS WG	AVR AVP MVR MVP TVP

Kim	2011	South Korea	2006–2009	R	26 ± 13.3	199	82	CY	PVI, PW, MI, LAA, MM, CS	AVR AVP MVR MVP TVP CABG ASD VSD

Breda	2011	Brazil	2003–2009	R	12.16 ± 10.89	15	15	RF	PVI, PW, MI, LAA, MM	MVR MVP

Albage	2011	Sweden	2005–2010	P, non-RCT	1–12R	44	71	CY	PVI, PW, MI, LAA, Maze III	AVR CABG MVP TVP MVR ASD MVP

McCarthy	2010	USA	2004–2008	P, non-RCT	5–24R	91	75	RF, CY, cut-and-sew	PVI LAA RAA TC	AVR MVR TVP CABG

Deneke	2009	Germany	NR	R	55 ± 17	64	66	RF	PVI, PW, MI, LAA, Maze III	MVR, AVR, CABG

Wang	2009	China	2004–2007	P, RCT	28 ± 5	150	149	RF	PVI, PW, MI, LAA, CTI, MM	MVR AVR MVP TVR TVP AVP

Srivastava	2008	India	NR	P, RCT	44	40	40	RF, CY	PVI, PW, MI, LAA, Maze III	MVR AVR MVP TVR TVP AVP

Geuzebroek	2008	Netherlands	1999–2005	R	NR	26	40	RF	PVI, PW, MI, LAA, Maze III	MVR AVR MVP TVR TVP AVP

Deneke	2007	Germany	1997–2005	R	21	106	116	RF	PVI MV LAA RAA, MM	MVR MVP CABG AVR

Calo	2006	Italy	NR	P, RCT	15 ± 5 (BA)/13 ± 6 (LA)	39	41	RF	PVI MV LAA RAA TC CS WG	NR

Ryan	2004	USA	1996–2003	R	595 ± 750 days	36	7	RF, CY, cut-and-sew	PVI, PW, MI, LAA, Maze III	NR

Guden	2003	Turkey	2001	P, non-RCT	10.9 ± 5.58	48	57	RF	PVI, PW, MI, LAA, Maze III	AVR CABG MVP TVP MVR ASD MVP

Takami	1999	Japan	NR	R	34.1 ± 11.3 (BA)/17.8 ± 3.8 (LA)	30	20	CY, cut-and-sew	PVI, PW, MI, LAA, CTI, Maze III	MVR, CABG, AVR, TVR

MO: month; R: range; P: p retrospective observational; RCT: randomized, controlled trial; BA: biatrial ablation; LA: left atrial ablation; CY: cryoablation; RF: radiofrequency ablation; MW: microwave ablation; PVI: pulmonary vein isolation; LAA: left atrial appendage; RAA: right atrial appendage; TC: terminal crest; CS: coronary sinus; WG: Waterston's groove; MI: mitral isthmus; MM: modified maze; CI: cavotricuspid isthmus; PW: posterior wall; AVR: aortic valve replacement; AVP: aortic valvuloplasty; MVR: mitral valve replacement; MVP: mitral valvuloplasty; TVP: tricuspid valvuloplasty; CABG: coronary artery bypass grafting; ASD: atrial septal defect repair; VSD: ventricular septal defect repair; NR: no report.

**Table 3:** Baseline characteristics of the included studies.

First author	Age (BA/LA, years)	Male (BA/LA)	Diabetes (BA/LA)	Heart failure (BA/LA)	Cerebrovascular events (BA/LA)	Hypertension (BA/LA)	Type of AF
Gualis	65.1 ± 10.2/71.6 ± 6.8	29/39	15/17	32/31	7/12	NR	Permanent persistent
Liu	49.87 ± 8.96/47.98 ± 8.64	21/23	NR	NR	NR	NR	Permanent persistent
Henn	64 ± 12	NR	NR	NR	NR	NR	Permanent persistent
Pecha	70.5 ± 7.3/70.1 ± 7.5	45/40	16/13	NR	NR	NR	Paroxysmal persistent permanent
Jiang	52.7 ± 4.9/50.7 ± 5.9	22/21	7/8	36/29	2/2	11/8	Paroxysmal persistent permanent
Soni	NR	NR	NR	NR	NR	NR	Paroxysmal persistent permanent
Pecha	59 ± 28/68 ± 12	54/116	16/40	NR	NR	67/118	Paroxysmal persistent permanent
Onorati	64 ± 9/65 ± 8	79/18	40/10	NR	NR	37/15	Permanent persistent
Kim	56.3 ± 12.0/52.1 ± 11.9	75/47	19/5	NR	NR	32/21	Paroxysmal persistent permanent
Breda	60.0 ± 8.07/46.3 ± 9.54	9/5	NR	10/6.	NR	NR	Permanent persistent
Albage	64.9 ± 10.4/66.9 ± 6.7	34/54	4/11	17/28	2/7	10/27	Paroxysmal persistent permanent
McCarthy	68.7 ± 10.3/66.8 ± 12.1	42/88	13/22	NR	NR	NR	Paroxysmal persistent permanent
Wang	67 ± 8/69 ± 9	NR	NR	NR	NR	NR	Permanent persistent
Deneke	53.4 ± 10.8/54.2 ± 10.1	54/62	NR	NR	NR	NR	Permanent persistent
Srivastava	37.11 ± 11.12/36.03 ± 7.99	19/22	NR	NR	NR	NR	Permanent persistent
Geuzebroek	63.3 ± 7.9/61.1 ± 10.3	21/17	NR	NR	NR	NR	Paroxysmal persistent permanent
Deneke	68 ± 9	NR	NR	NR	NN	NR	Paroxysmal persistent permanent
Calo	57.9 ± 8.9/59.2 ± 9.1	26/26	NR	NR	NR	16/18	Paroxysmal persistent permanent
Ryan	NR	NR	NR	NR	NR	NR	Paroxysmal persistent permanent
Guden	52 ± 11/54 ± 9	14/23	NR	NR	N	NR	Permanent persistent
Takami	54.7 ± 8.8/58.3 ± 8.7	11/9	NR	NR	NR	NR	Paroxysmal persistent permanent

BA: biatrial ablation; LA: left atrial ablation; NR: no report.

**Table 4:** Sensitivity and subgroup analyses.

Endpoint	Restored SR at discharge		Restored SR at 12 months		Restored SR beyond 1 year	
	Overall *P* value	OR (95% CI)	Overall *P* value	OR (95% CI)	Overall *P* value	OR (95% CI)
*Study design*					0.46	1.37 (0.60, 3.11)
RCT	0.38	0.51 (0.12, 2.25)	Have no RCT studies		0.69	1.25 (0.43, 3.65)
Non-RCT	0.4	1.17 (0.81, 1.68)				
*Study size*						
<150	0.88	1.05 (0.56, 1.95)	0.04	1.76 (1.02, 3.04)	0.76	1.22 (0.24, 4.46)
>150	0.96	0.99 (0.66, 1.49)	0.44	1.29 (0.67, 2.48)	0.3	1.44 (0.73, 2.85)
*Statistical models*						
Fixed-effect	0.3	1.12 (0.90, 1.38)	0.009	1.39 (1.09, 1.79)	0.16	1.21 (0.92, 1.59)
Random-effect	0.34	1.15 (0.86, 1.55)	0.2	1.37 (0.85, 2.21)	0.4	1.31 (0.70, 2.48)

SR: sinus rhythm; RCT: randomized, controlled trial.
